# Atypical Presentation of Glioblastoma: A Case Report

**DOI:** 10.7759/cureus.72160

**Published:** 2024-10-22

**Authors:** Tambi Isaac, Nassir Mansour

**Affiliations:** 1 Surgery, Wyckoff Heights Medical Center, New York, USA; 2 Medical Academy, Kabardino-Balkarian State University, Nalchik, RUS; 3 Neurological Surgery, Wyckoff Heights Medical Center, New York, USA

**Keywords:** astrocytoma, brain tumor, cns tumors, glioblastoma, glioma

## Abstract

This case report describes a patient who presented with devastating stroke-like symptoms secondary to a cystic brain lesion that was confirmed to be glioblastoma without significant symptomatology before her dramatic presentation. It further highlights the aggressive nature and the swift growth of the tumor in a short period of time.

A 55-year-old female with no significant past medical history presented to the emergency department in a comatose state. The patient had experienced a week of worsening headaches and vomiting, self-medicating with Tylenol. Upon evaluation, she was noticed to have bilaterally dilated fixed pupils: R: 6 mm, L: 3 mm. The initial head CT revealed a right frontal cystic lesion associated with descending trans-tentorial brain herniation and brain-stem compression. Because of her relatively young age and sudden onset symptoms, the decision was made to take her urgently to the operating room. The patient underwent an emergency right frontal craniotomy, resection, and lobectomy. The patient was intubated and ventilated after surgery. The postoperative exam was significant for pupils ~2-3 mm and reactive to light. On postoperative day 1, the patient became very responsive and was eventually extubated. She was found to have a normal neurological examination after extubating her; despite the devastating presentation and extent of the tumor, the patient recovered well without significant motor or behavioral deficits. The surgical intervention prevented an impending brain death. A follow-up MRI after two weeks shows a substantial recurrence of the tumor as compared to imaging upon discharge. The patient was taken again to the operating room for a second surgery. She was discharged a few days after her second operation with follow-up recommendations with oncology.

This case is among very few cases that highlight a swift and dramatic presentation of glioblastoma and isocitrate dehydrogenase-wild type grade IV in a patient without precipitating symptoms succeeding the main presentation. It also emphasizes rapid approaches to prevent a dreadful prognosis in this well-known aggressive type of brain tumor.

## Introduction

Brain tumors can be classified as primary (the tumor starts in the brain) or secondary (the tumor metastasizes to the brain) [1]. Brain tumors are graded on a four-point scale (1-4). Grade 1 is the least severe, and grade 4 is the most aggressive. Primary brain tumors are divided into gliomas, meningiomas, pituitary adenomas, and nerve sheath tumors. Gliomas are classified into different types based on the cell of origin of the tumor. In our case, we will talk about astrocytoma and glioblastoma

Astrocytoma is a type of glioma [2] originating from the brain's glial cells (hence the name glioma). These tumors are further divided based on a mutation of an enzyme called isocitrate dehydrogenase (IDH) that is found in their cells. The mutation is associated with a more favorable prognosis. When astrocytoma is grade 4 and lacks a mutation in the IDH enzyme, it is called glioblastomas, previously known as glioblastoma multiforme. These tumors are notoriously known to be more aggressive and have the worst prognosis compared to astrocytomas IDH mutants [3].

The presentation of glioblastoma may vary and depends largely on the size and location of the tumor [4]. Symptoms include but are not limited to headaches, personality changes, nausea, mood changes, and stroke-like symptoms. These symptoms usually develop gradually over time in concordance with the tumor growth. In our case, the patient did not have significant proceeding symptoms prior to the main presentation, which makes this an unusual presentation of glioblastoma and gives a sense of the rapid growth nature of this tumor.

Causality of glioblastoma is associated with DNA mutations like other cancers; however, it remains not fully clear. Tumor cells originate from neural glial cells that support nerves. More recent studies suggest that astrocytes, oligodendrocyte progenitor cells, and neural stem cells could all serve as the cells of origin [5].

## Case presentation

This case presents a 55-year-old lady with no significant past medical history who was brought by ambulance to a local hospital in New York in an unconscious state. The patient was accompanied by her family, who provided collateral history.

This relatively young lady was healthy overall with no past medical history or alarming symptoms. She was taking Tylenol for an occasional headache that started about two days ago, which she attributed to stress. There were no records of previous hospitalizations and nearly no significant symptoms before this.

On the morning of the day of the presentation, our patient experienced a debilitating decline in consciousness; while lying in bed, she was unresponsive to her husband's attempts to arouse her. Shortly after, she started vomiting several times and appeared drowsy and unable to open her eyes fully. The lady was rushed to the emergency department by ambulance, and en route, she was intubated due to deterioration of her consciousness.

**Figure 1:**
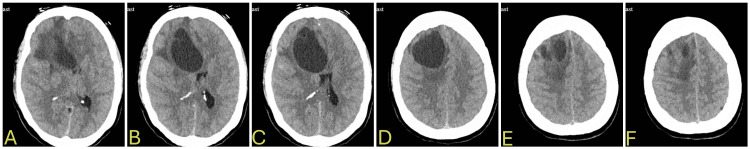
Brain CT during initial encounter A-F: right frontal cystic mass suggestive of glioblastoma with midline shift and brain stem compression CT: computed tomography

The physical exam was significant for comatose state GCS 4, right pupil 6 mm nonreactive, left pupil 3 mm nonreactive, no tracking, no corneal, cough, or gag reflexes, and no spontaneous or any movements to noxious stimuli in all extremities. A CT scan of the brain showed a right frontal cystic mass with midline shift and compression, as shown in Figure 1.

The patient was taken to the operating room urgently for right frontal craniotomy with resection and decompression of the right frontal cystic lesion in addition to right frontal lobectomy, without which the patient may have suffered an unreversible brain injury instantly.

Narrative

**Figure 2:**
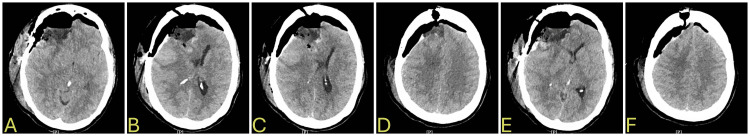
Day 1 brain CT after lesion resection and lobectomy A-F: improvement in the right-to-left midline shift and the mass effect on the ventricles CT: computed tomography

**Figure 3:**
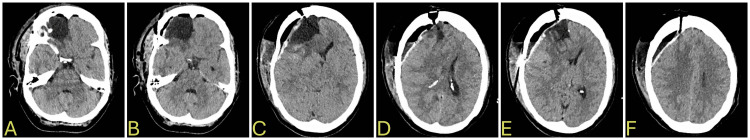
Day 2 brain CT after lesion resection and lobectomy A-F: continuous improvement in the mass effect and reduced swelling two days after lesion resection and lobectomy CT: computed tomography

**Figure 4:**
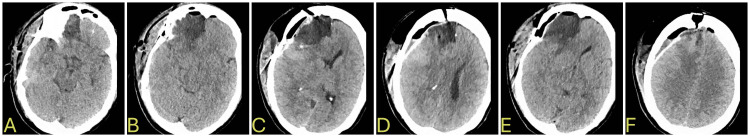
Day 3 brain CT after lesion resection and lobectomy A-F: continuous improvement of the mass effect CT: computed tomography

The patient underwent emergent surgery on the same day of presentation, and she was transferred to the ICU. A postoperative serial CT scan on days one, two, and three showed improvement in the right to left midline shift and the mass effect on the ventricles, as shown below in Figures 2-4.

The patient was followed up by a team consisting of medicine, neurology, and neurosurgery. Postoperative medications included cefazolin, dexamethasone, levetiracetam, mannitol, pantoprazole, propofol, and natrium chloride.

The first postoperative physical exam showed pupils 2 mm reactive to light and accommodation, positive corneal reflex oculus uterque, positive gag and cough reflexes, and positive response (grimaces) to painful stimuli. On postoperative day 2, the patient was weaned off sedation and extubated after confirming spontaneous respiratory effort. She was able to open her eyes and follow commands (Glasgow Coma Scale (GCS) 15). Weakness was more pronounced in the proximal upper extremities versus the lower. The right side was normal. On the fourth day, the patient returned almost to baseline strength with no neurological deficits noticed at all, which was remarkably exceptional. She was discharged home with a recommendation to follow up in the clinic. Discharge medications included acetazolamide 125 mg every eight hours, acetaminophen 500 mg every six hours, oxycodone 5 mg every eight hours, dexamethasone 1 mg daily on tapering dose, and levetiracetam 750 mg every 12 hours.

**Figure 5:**
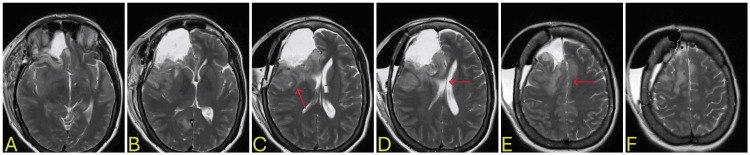
Day 4 brain MRI after lesion resection and lobectomy and prior to discharge A-F: postoperative changes in the right frontal region, D-E: area of swelling and midline shift (red arrow) MRI: magnetic resonance imaging

MRI prior to discharge showed postoperative changes in the right frontal region with a large cystic lesion (Figure 5). Persistent mass effect and midline shift measure 1 cm from right to left compared to 1.4 cm on preoperative CT.

During a follow-up visit to the clinic after two weeks, the patient presented with a relatively large pseudomeningocele with a CSF leak. The patient was presented with two options: one is to wait and watch with an increased dosage of acetazolamide, and the other is to be admitted and plan an abdominal fat graft and dural repair with a small chance of intraperitoneal shunting in case of recurrence as a last resort. The patient opted for the surgical option.

**Figure 6:**
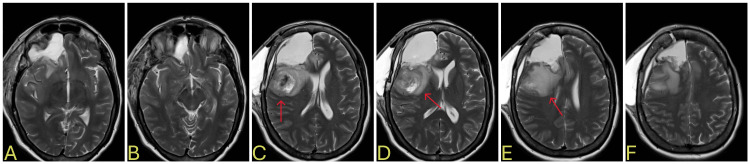
Brain MRI prior to the second admission, 19 days after the first resection A-B: postoperative changes and swelling 19 days after the first resection, C-F: a new mass formation that was not seen on previous MRI with midline shift (red arrow), significant enhancement at the lateral aspect of the resection cavity MRI: magnetic resonance imaging

Following her readmission, another MRI was done and showed the development of a new mass in the right frontal-parietal lobe measuring 34 mm with overlying cerebral edema and 7 mm right to left midline shift (Figure 6). Diffuse abnormal enhancement in the region of the resection site in the area of the mass in the right frontal-parietal lobe concerns for recurrence with extension into the subdural space. When compared to the MRI from two weeks ago following discharge from the hospital, a significant enhancement at the most lateral inferior part of the resection cavity was noticed. Based on the MRI results, a wound exploratory surgery was planned to resect the recurring tumor.

Following surgery, the patient was monitored for four days and was discharged home on the fifth day. Her physical exam was normal, and she remained without neurological deficits or changes in her behavior. Pupils were equal to 3 mm and reactive to light. Motor strength is 5/5 overall and reflexes preserved +2. There were no signs of upper motor neuron lesions. Palmar drift and Hoffman's sign were negative throughout the recovery period and after.

**Figure 7:**
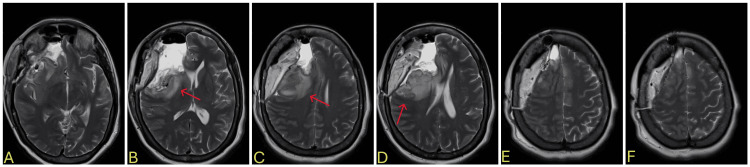
Day 2 brain MRI after the second surgery (wound exploration and resection of the recurrence) A-F: postoperative area of enhancement with overlying cerebral edema (red arrow) MRI: magnetic resonance imaging

Brain MRI on day 2 after the resection of the recurrent tumor shows enhancement in the postoperative area with overlying cerebral edema and midline shift (Figure 7). Diffuse peripheral rim enhancement extends to the right frontal subdural space.

The patient was instructed to start radiation and chemotherapy and follow up with us at the outpatient clinic to monitor her case. Discharge medication included acetaminophen, acetazolamide 125 mg every eight hours, and levetiracetam 750 mg every 12 hours.

## Discussion

Glioblastoma accounts for 14.2% of all tumors and 50.9% of all malignant tumors, with an incidence rate of 3.27 per 100,000 population, which is the highest among malignant tumors [6]. The median age of diagnosis is 66 years, and men are more commonly affected than women [6]. The prognosis and survival rate are quite low, with 40% of patients surviving the first year post-diagnosis and 17% surviving the second year. Median survival time is about 15 months [7]. However, long survival cases have been reported, with one of the longest survival cases being 26 years after the treatment [8].

The presentation of glioblastoma depends on location and size, as we have demonstrated above. In our case, the presentation was quite atypical, with the patient margining from being totally asymptomatic or mildly symptomatic with only occasional headaches for a couple of days to the most devastating stroke-like presentation in a matter of hours. Compared to other reported cases of newly diagnosed glioblastoma, many patients present with seizures and evolving neurologic deficits several weeks before the diagnosis, as highlighted in the review by Johnson et al. [9].

In addition, glioblastoma is known for its rapid growth, which was evident in our case, as the residual tumor regrew within just two weeks following the initial surgery. This phenomenon was observed in many several case reports of glioblastoma [10]. A hypothesis exists that the low survival rate may be related to the high proliferation rate and aggressive growth of the tumor and that the tumor cell doubling may predict survival time. A previous study reported that the tumor cell doubling time ranges from two days to several weeks [10].

While MRI remains a crucial method to diagnose glioblastoma, we know now that glioblastoma is associated with IDH wild type. This may be useful in developing new methods of diagnosis and treatment where the specific cells may be captured and targeted or at least slowed down if the previously mentioned theory is deemed to be a fact.

## Conclusions

This case highlights the aggressive nature of glioblastoma, IDH wild type, CNS WHO grade 4, with its ability to progress rapidly and often without warning. It reinforces the need for prompt surgical intervention to mitigate the risk of fatal outcomes. The unexpectedly favorable recovery in our patient demonstrates that early and aggressive treatment can positively impact prognosis, even in severe presentations.

Close and frequent follow-up remains essential to monitor for recurrence. Looking ahead, emerging therapies such as immunotherapy, oncolytic virotherapy, and vaccine therapy show promise and may complement standard treatments like surgery, chemotherapy, and radiation. These novel approaches offer new hope in the fight against glioblastoma and could reshape treatment strategies, potentially improving outcomes for many patients facing this devastating disease.
